# “In-Group” Communication in Marine *Vibrio*: A Review of N-Acyl Homoserine Lactones-Driven Quorum Sensing

**DOI:** 10.3389/fcimb.2018.00139

**Published:** 2018-05-07

**Authors:** Jianfei Liu, Kaifei Fu, Chenglin Wu, Kewei Qin, Fei Li, Lijun Zhou

**Affiliations:** Central Laboratory, Navy General Hospital of Chinese People's Liberation Army, Beijing, China

**Keywords:** N-acyl homoserine lactone, quorum sensing (QS), *Vibrio*, pathogenicity, intervention

## Abstract

N-Acyl Homoserine Lactones (N-AHLs) are an important group of small quorum-sensing molecules generated and released into the surroundings by Gram-negative bacteria. N-AHLs play a crucial role in various infection-related biological processes of marine *Vibrio* species, including survival, colonization, invasion, and pathogenesis. With the increasing problem of antibiotic abuse and subsequently the emergence of drug-resistant bacteria, studies on AHLs are therefore expected to bring potential new breakthroughs for the prevention and treatment of *Vibrio* infections. This article starts from AHLs generation in marine *Vibrio*, and then discusses the advantages, disadvantages, and trends in the future development of various detection methods for AHLs characterization. In addition to a detailed classification of the various marine *Vibrio*-derived AHL types that have been reported over the years, the regulatory mechanisms of AHLs and their roles in marine *Vibrio* biofilms, pathogenicity and interaction with host cells are also highlighted. Intervention measures for AHLs in different stages are systematically reviewed, and the prospects of their future development and application are examined.

Quorum Sensing (QS) is a phenomenon that allows bacterial communities to sense small auto-secreting molecules in the environment, allowing monitoring of population density and then regulating expressions of related genes (Bassler, 1999). These small molecules involved in bacterial QS, also known as AutoInducers (AIs) (Nealson, 1977), are classified into three types based on their synthesis pathways, namely AutoInducer-1 (AI-1), AutoInducer-2 (AI-2), and AutoInducing Peptides (AIPs) (Williams, 2007). Different bacterial species generate different AIs to carry out their QS-dependent regulatory functions.

Gram-negative bacteria can mainly produce AI-1 and AI-2 signaling molecules that mediate QS signal transduction via different pathways (Mok et al., 2003; Liaqat et al., 2014). For example, they produce N-Acyl Homoserine Lactones (N-AHLs, AI-1) to mediate QS, to regulate various functions such as biofilm formation, toxin expression, and to escape from host immune response. Increasing studies on the role and underlying mechanism of AHLs in recent years revealed that AHLs are closely associated with the survival and the pathogenicity of most bacteria (Horng et al., 2002; Lumjiaktase et al., 2006; García-Aljaro et al., 2012a).

*Vibrio* are Gram-negative bacteria commonly found in the marine environment, and 12 of them have been reported as marine pathogen (Balows et al., 1991). They are not only pathogenic to many animal species used in the aquaculture industry, but also are responsible for a number of human gastrointestinal, wound, and even severe acute infections (Tarr et al., 2015). Since QS is common among marine *Vibrio*, understanding the generation, characteristics, functional regulation, and intervention means of AHLs will help increase knowledge not only on the species but also on the prevention and treatment of infections caused by *Vibrio*. This article provides an overview of the current progress and knowledge gaps on the generation characteristics, detection, regulatory functions of AHLs in marine *Vibrio*, and several different AHL-related intervention measures as well.

## Characteristics and detection of marine *Vibrio* AHLs

**Figure 1:**
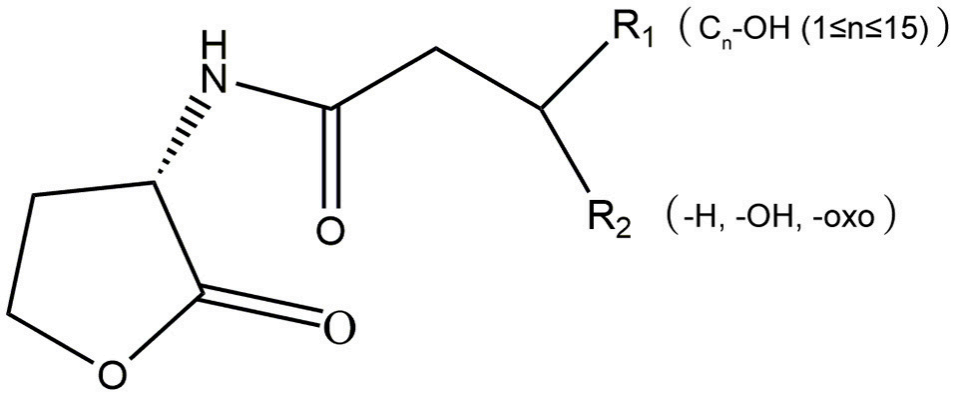
The molecular structure of AHLs. **R1**: one at least and 15 at most are included from the 4th carbon atom on the acyl side chain of AHL molecule; **R2**: the unsubstituted (-H) or substituent groups (-OH, -oxo) on the 3rd carbon of the acyl side chain.

AHLs are a group of amphipathic small molecules (Figure 1), and their common structure is comprised of a hydrophilic homoserine lactone ring and a hydrophobic acyl side chain (O'Connor et al., 2015). Differences in molecular structures depend on the number of carbon (4–18), the substituent group on the third carbon (-H, -OH or -oxo), and the presence or absence of unsaturated double bonds in the acyl side chains (Kumari et al., 2006). These differences cause the diversity in the molecular structures of AHLs and in their secretion pathways. While short side-chain AHLs (<8 carbon atoms on acyl side chain, C_4−8_-HSL) can directly penetrate cell membrane and be released into the surrounding environment upon synthesis, long side-chain AHLs (>8 carbons on acyl side chain, C_10−18_-HSL) on the contrary can only be released through active efflux pathways, such as 3-oxo-C_12_-HSL being exported from membranes via an active *mexAB-oprM-*encoded MexAB-OprM pump (Pearson et al., 1999). Therefore, diversity of AHLs not only indicates differences in the application of detection methods, but also serves as the basis for various functional regulation.

**Figure 2:**
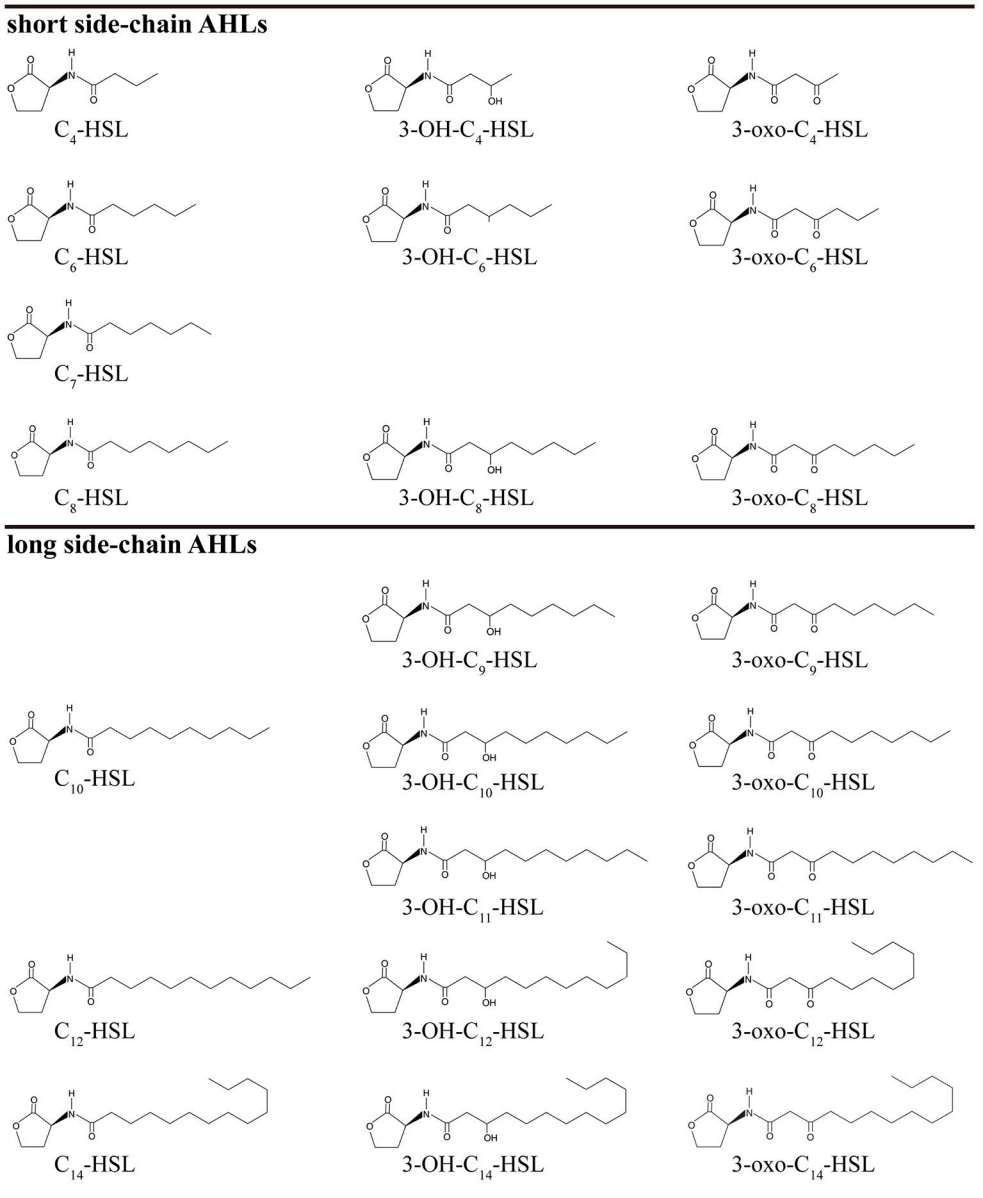
Chemical structure of 23 AHLs produced by marine *Vibrio*.

AHLs being generated by *Vibrio* species and AHL types being accurately detected are two important questions in QS-related studies in marine *Vibrio*. Common detection methods for AHLs include microbiosensor-based biological detection and chromatography/Mass Spectrometry (MS)-based physicochemical detection. Based on current literatures, a total of 32 AHLs-producing marine *Vibrio* species have already been identified using different detection methods. Out of the 32, 23 AHLs were definitely classified, including 10 short side-chain and 13 long side-chain AHLs (Figure 2;Tables 1,2).

**Table 1:** Statistics of the AHL types and detection methods produced by marine *Vibrio*.

***Vibrio* spp**.	**C**_**n**_**-HSL**							**3-OH-C**_**n**_**-HSL**								**3-oxo-C**_**n**_**-HSL**								**References**
	**4**	**6**	**7**	**8**	**10**	**12**	**14**	**4**	**6**	**8**	**9**	**10**	**11**	**12**	**14**	**4**	**6**	**8**	**9**	**10**	**11**	**12**	**14**	
*V. aestuarianus*				+^a^														+^a^						Yang et al., 2011; Garcia-Aljaro et al., 2012b
*V. anguilarum*	+^b^	+^b^^a^		+^d^^a^				+^a^	+^d^^b^^a^	+^b^^a^		+^d^^b^				+^d^^a^	+^a^	+^d^^a^		+^d^^b^^a^		+^d^^b^^a^		Milton et al., 1997, 2001; Buch et al., 2003; Buchholtz et al., 2006; Garcia-Aljaro et al., 2012b; Purohit et al., 2013; Rasmussen et al., 2014
*V. brasiliensis*	+^d^	+^d^^b^						+^d^												+^b^				Rasmussen et al., 2014; Tan W. S. et al., 2014
*V. campbellii*	+^a^	+^a^^e^				+^d^		+^d^	+^d^					+^d^			+^a^^e^							Taylor et al., 2004; Tait et al., 2010; Rasmussen et al., 2014
*V. coralliilyticus*	+^a^																							Tait et al., 2010
*V. fischeri*		+^b^		+^f^													+^a^^f^^h^^i^	+^a^						Eberhard et al., 1981; Kuo et al., 1994; Shaw et al., 1997
*V. fluvialis*		+^a^		+^d^^a^	+^g^							+^d^	+^d^			+^d^		+^d^	+^d^	+^d^^g^	+^d^	+^d^^g^		Yang et al., 2011; Wang et al., 2013; Rasmussen et al., 2014
*V. furnissii*				+^a^								+^a^		+^a^				+^a^						Yang et al., 2011; Viswanath et al., 2015
*V. gaogenes*		+^a^																						Yang et al., 2011
*V. harveyi*	+^a^																							Tait et al., 2010; Garcia-Aljaro et al., 2012b
*V. mediterranei*				+^a^													+^a^					+^a^		Taylor et al., 2004; Yang et al., 2011; Garcia-Aljaro et al., 2012b
*V. metschnikovii*				+^a^																		+^a^		Garcia-Aljaro et al., 2012b
*V. pacinii*	+^d^							+^d^																Rasmussen et al., 2014
*V. proteolyticus*				+^a^												+^a^								Yang et al., 2011; Garcia-Aljaro et al., 2012b; Viswanath et al., 2015
*V. rotiferianus*	+^d^^a^	+^d^	+^d^	+^d^^a^																				Tait et al., 2010; Garcia-Aljaro et al., 2012b; Rasmussen et al., 2014
*V. salmonicida*		+^a^^b^															+^a^^b^							Bruhn et al., 2005
*V. scophthalmi*														+^b^										García-Aljaro et al., 2008
*V. shiloi*	+^a^																							Tait et al., 2010
*V. sinaloensis*	+^b^																							Tan P. W. et al., 2014
*V. splendidus*	+^b^	+^d^^b^	+^d^	+^a^				+^d^^b^	+^d^^b^	+^b^	+^d^	+^d^		+^d^	+^d^									Garcia-Aljaro et al., 2012b; Purohit et al., 2013; Rasmussen et al., 2014
*V. tasmaniensis*	+^a^				+^c^		+^c^							+^c^								+^c^		Tait et al., 2010; Girard et al., 2017
*V. tubiashii*		+^d^	+^d^					+^d^	+^d^							+^d^								Rasmussen et al., 2014
*V. vulnificus*	+^b^	+^b^																+^b^		+^b^		+^b^	+^b^	Morin et al., 2003; Valiente et al., 2009; Garcia-Aljaro et al., 2012b
*V. xiamenensis*		+^a^		+^a^								+^a^		+^a^										Viswanath et al., 2015

+, detectable;

a , TLC-biosensor;

b , HPLC-MS;

c , UHPLC-MS;

d , UHPLC-DAD-QTOFMS;

e , GC-MS;

f , NMR;

g , ESI-MS;

h , IS;

i *, FRET*.

**Table 2:** Chemical structure information of AHLs produced by marine *Vibrio*.

**AHL name**	**Abbreviation**	**Molecular formula**	**Molecular weight**
N-Butyryl-DL-homoserine lactone	C_4_-HSL	C_8_H_13_NO_3_	171.2
N-Hexanoyl-L-homoserine lactone	C_6_-HSL	C_10_H_17_NO_3_	199.2
N-heptanoyl-L-homoserine lactone	C_7_-HSL	C_11_H_19_NO_3_	213.3
N-Octanoyl-L-homoserine lactone	C_8_-HSL	C_12_H_21_NO_3_	227.3
N-Decanoyl-DL-homoserine lactone	C_10_-HSL	C_14_H_25_NO_3_	255.4
N-Dodecanoyl-DL-homoserine lactone	C_12_-HSL	C_16_H_29_NO_3_	283.4
N-Tetradecanoyl-DL-homoserine lactone	C_14_-HSL	C_18_H_33_NO_3_	311.5
N-(3-Hydroxybutyryl)-L-homoserine lactone	3-OH-C_4_-HSL	C_8_H_13_NO_4_	187.2
N-(3-Hydroxyhexanoyl)-L-homoserine lactone	3-OH-C_6_-HSL	C_10_H_17_NO_4_	215.2
N-(3-Hydroxyoctanoyl)-DL-homoserine lactone	3-OH-C_8_-HSL	C_12_H_21_NO_4_	243.3
N-(3-Hydroxynonanoyl)-L-Homoserine lactone	3-OH-C_9_-HSL	C_13_H_23_NO_4_	257.3
N-(3-Hydroxydecanoyl)-L-Homoserine lactone	3-OH-C_10_-HSL	C_14_H_25_NO_4_	271.4
N-(3-Hydroxyundecanoyl)-L-Homoserine lactone	3-OH-C_11_-HSL	C_15_H_27_NO_4_	269.4
N-(3-Hydroxydodecanoyl)-DL-homoserine lactone	3-OH-C_12_-HSL	C_16_H_29_NO_4_	299.4
N-(3-Hydroxytetradecanoyl)-DL-homoserine lactone	3-OH-C_14_-HSL	C_18_H_33_NO_4_	327.4
N-(3-Oxoburtyryl)-L-homoserine lactone	3-oxo-C_4_-HSL	C_8_H_11_NO_4_	185.2
N-(β-Ketocaproyl)-DL-homoserine lactone	3-oxo-C_6_-HSL	C_10_H_15_NO_4_	213.2
N-(3-Oxooctanoyl)-L-homoserine lactone	3-oxo-C_8_-HSL	C_12_H_19_NO_4_	241.3
N-(3-Oxononanoyl)-L-homoserine lactone	3-oxo-C_9_-HSL	C_13_H_21_NO_4_	255.3
N-(3-Oxodecanoyl)-L-homoserine lactone	3-oxo-C_10_-HSL	C_14_H_23_NO_4_	269.3
N-(3-Oxoundecanoyl)-L-homoserine lactone	3-oxo-C_11_-HSL	C_15_H_25_NO_4_	283.3
N-(3-Oxododecanoyl)-L-homoserine lactone	3-oxo-C_12_-HSL	C_16_H_27_NO_4_	297.4
N-(3-Oxotetradecanoyl)-L-homoserine lactone	3-oxo-C_14_-HSL	C_18_H_31_NO_4_	325.4

### Characteristics of AHLs generation in marine *Vibrio*

AHLs generation differs among different marine *Vibrio* species (Eberhard, 1972; Nealson, 1977). For example, *V. anguillarum* produces more than 12 types of AHLs (Milton et al., 1997, 2001; Buch et al., 2003; Buchholtz et al., 2006; Purohit et al., 2013; Rasmussen et al., 2014), whereas *V. scophthalmi* and *V. harveyi* only produce one type of detectable AHL (Tait et al., 2010; Garcia-Aljaro et al., 2012b), indicating that the number of AHLs generated largely varies among marine *Vibrio*. Furthermore, only long side-chain AHLs have so far been detected in *V. scophthalmi* (García-Aljaro et al., 2008). In contrast, only short side-chain AHLs are produced in 12 *Vibrio* spp., such as *V. tubiashii* and *V. fischeri* (Eberhard et al., 1981; Kuo et al., 1994; Shaw et al., 1997; Rasmussen et al., 2014), further indicating that the AHL types and proportions also largely differ among marine *Vibrio*.

AHLs generation is also significantly different between various strains of the same species (Greenberg et al., 1979). Tait et al. (2010) isolated several strains of *V. campbelii* from coral-associated *Vibrio* (Tait et al., 2010), and found out that the AHLs detected and identified in the different strains of *V. campbelii* varied significantly, indicating that AHL generation is diverse and complex even within the same environment. This pattern of AHL generation may be associated with the rapid adaptation of *Vibrio* to environmental changes (Persat et al., 2014).

The composition of AHLs generated by marine *Vibrio* is significantly different from those found in terrestrial bacteria. Apart from the AHLs that are commonly generated in terrestrial bacteria, marine *Vibrio* generate many types of ultra-long side-chain AHLs, such as C_14_-HSL (Girard et al., 2017), 3-OH-C_14_-HSL (Rasmussen et al., 2014), and 3-oxo-C_14_-HSL (Morin et al., 2003). On the other hand, AHLs such as C_7_-HSL, 3-OH-C_9_-HSL, 3-oxo-C_9_-HSL, 3-OH-C_11_-HSL, and 3-oxo-C_11_-HSL are rarely identified or reported in terrestrial bacteria, but are also detected in marine *Vibrio* (Rasmussen et al., 2014).

The environmental conditions that induce generation of AHLs in marine *Vibrio* are also different from those required by terrestrial bacteria. Firstly, the optimum temperature needed in marine *Vibrio* is lower than that of common terrestrial bacteria to produce AHLs. In fact, marine *Vibrio* produce more types and higher concentrations of AHLs at lower temperatures (< 16°C). Thus, the AHLs diversity and concentration decrease with increasing temperature (Tait et al., 2010). Secondly, marine *Vibrio*-derived AHL types are affected more by changes in ion levels than those generated by common terrestrial bacteria (Buchholtz et al., 2006), which may be associated with greater seasonal variation in temperature and ion levels in marine environment because of complex ocean hydrography. In addition, the dominant AHL alters as the colonization state of marine *Vibrio* changes, and no report has been described by evidence on this alteration in dominant AHL in terrestrial bacteria to this day. For example, when free *V. anguillarum* infects the host, its dominant AHL changes from 3-oxo-C_10_-HSL to 3-OH-C_6_-HSL (Buchholtz et al., 2006). This change in dominant AHL types could be associated with the various regulatory mechanisms in which AHLs are involved.

### Biological detection of AHLs

Previously, AHLs generation was measured indirectly by real-time monitoring of bacterial growth rate and AHL-related gene expression, which are time and energy consuming, and has low efficiency and poor accuracy (Bainton et al., 1992; Pearson et al., 1994). With the increasing understanding of the AHL-QS regulatory mechanisms, microbial-derived biosensors gradually replaced the above detection methods and became the conventional and standard technique for AHLs identification (O'Connor et al., 2015). Microbiosensors lack AHLs synthesis proteins but still contain the related AHL receptor proteins and functional genes. Under exogeneous AHLs stimulation, the expression of reporter genes can be initiated, which are then reflected by the changes in colony color, luminescence or enzyme activities. Microbiosensors are mainly obtained in two ways: (1) natural environmental mutation, and (2) genome editing.

For the mutation, although bacterial strains can no longer synthesize AHLs and have lost the characteristic functional expression due to gene mutation, they are still able to initiate QS regulation via exogenous AHLs recognition, leading to characteristic changes in pigments, bioluminescence and protease activities. An example of this type of biosensor is *Chromobacterium violaceum* CV026, which is a mini-Tn5 mutant of *C. violaceum* ATCC31532. *C. violaceum* CV026 has lost the ability to synthesize purple pigments itself but can proliferate purple colonies under exogenous AHLs stimulation. It is highly sensitive to short side-chain AHLs without substituents on the 3rd carbon of the acyl side chain, and with C_6_-HSL as the AHL having the strongest activating capability. Furthermore, its sensitivity to short side-chain AHLs is decreased by approximately 10-fold when carbonyl substituent is present on the 3rd carbon of the acyl side chains. In contrast, short side-chain AHLs with hydroxyl substituents on the 3rd carbon of the acyl side chain are not recognized by *C. violaceum* CV026 (McClean et al., 1997).

In the second type of microbiosensors construction, artificial plasmid insertion based on direct genome editing is carried out in bacterial cells to sense exogenous AHLs that induce reporter gene expression via the recombinant plasmid, leading to changes in biological characteristics of microbiosensors of this type. *Agrobacterium tumefaciens* KYC55 is a biosensor with broad-spectrum AHLs detection capacity acquired artificially, and is highly sensitive to long side-chain AHLs. *A. tumefaciens* KYC55 contains the p*T7-traR* plasmid, which has a p*tral-lacZ* promotor triggered by broad-spectrum AHLs to initiate the expression of the *lacZ* gene. The *lacZ* gene encodes β-galactosidase, which then hydrolyzes 5-bromo-4-chloro-3-indole-β-D-galactopyranoside (X-Gal) to produce a blue color in the bacterial colony (Zhu et al., 2003).

Given the diversity in microbiosensors, the types of detectable AHLs and their Limits of Detection (LOD) would also vary. Therefore, the detection of different AHLs generated by marine *Vibrio* would then be performed one by one across multiple microbiosensors for several times, with the concentrations of the generated AHLs being indirectly calculated. Although this method is not precise, its low cost and ease of use make it a popular technique for the crude screening of AHLs in marine *Vibrio*. Previously reported microbiosensors used for detecting AHLs generated by marine *Vibrio* are listed in Table 3.

**Table 3:** The biosensors and specific traits for the detection of AHLs produced by marine *Vibrio*.

**Microbiosensors**	**Plasmid**	**Sensing system**	**AHL types**	**Report system**	**Functional expression**	**References**
						**author, year**
*C. violaceum* CV026	—	CviI/RC	C_6_-HSL^*^; C_4_-HSL; C_8_-HSL; 3-oxo-C_4−8_-HSL	endogenous pigment	purple colony	McClean et al., 1997
*E. coli*	pSB 536	AhyI/R	C_4_-HSL^*^	LuxCDABE	bioluminescence	Tait et al., 2010
*E. coli*	pSB 401	LuxI/R	3-oxo-C_6_-HSL^*^; C_6_-HSL; C_8_-HSL	LuxCDABE	bioluminescence	Tait et al., 2010
*E. coli* MT102	pSB403	LuxI/R	3-oxo-C_6_-HSL^*^; C_6_-HSL; C_8_-HSL; 3-oxo-C_8_-HSL	LuxCDABE	bioluminescence	Charlesworth et al., 2015
*E. coli*	pSB 1075	LasI/R	3-oxo-C_12_-HSL^*^; C_12_-HSL; 3-oxo-C_10_-HSL	LuxCDABE	bioluminescence	Tait et al., 2010
*A. tumefaciens* NTL1	pDCI41E33	TraI/R	3-oxo-C_4_-HSL - 3-oxo-C_12_-HSL^*^; C_6_-HSL – C_12_-HSL	*lacZ*	β-galactosidase expression	O'Connor et al., 2015
*A. tumefaciens* NTL4	pZLR4	TraI/R	3-oxo-C_8_-HSL^*^; C_6−14_-HSL; 3-OH-C_6−10_-HSL; 3-oxo-C_4−14_-HSL	*lacZ*	β-galactosidase expression	Kumar et al., 2016
*A. tumefaciens* KYC55	pJZ372 pJZ384 pJZ410	TraI/R T7 T7	3-oxo-C_8_-HSL^*^; C_6−10_-HSL; 3-OH-C_6−10_-HSL; 3-oxo-C_6_-HSL; 3-oxo-C_12_-HSL	*lacZ*	β-galactosidase expression	Joelsson and Zhu, 2006
*P. putida* F117	pAS-C8	CepI/R	C_8_-HSL^*^; C_10_-HSL	*gfp*	green fluorescence	Steidle et al., 2001
*P. putida* F117	pKR-C12	LasI/R	3-oxo-C_12_-HSL^*^; 3-oxo-C_10_-HSL	*gfp*	green fluorescence	Krick et al., 2007

* *most sensitive AHLs*.

### Physicochemical detection of AHLs

Thin Layer Chromatography (TLC) is a type of Liquid-Solid Absorption Chromatography (LSAC) commonly used in combination with microbiosensors. Generally, AHL standards and test samples are loaded onto the TLC plate and immersed in the developing solution, causing samples to migrate with different speed. After the plate is dried, the culture media containing microbiosensor is then added onto the plate for culture. Color changes or luminescence in the colonization sites of the microbiosensor are used as the reporter signals for determining the types of AHL via comparison with the AHL standards (Huang et al., 2012). The TLC-biosensor combination is a cheap, rapid and highly efficient detection method that qualitatively and semi-quantitatively identifies the types and concentrations of AHLs in mixtures (Sun et al., 2010). This makes it a favored common preliminary screening technique for AHLs detection in marine *Vibrio*. Shaw et al. (1997) was the first to utilize the TLC-biosensor method to show the generation of 3-oxo-C_6_-HSL and 3-oxo-C_8_-HSL from *V. fischeri* (Shaw et al., 1997). In 2015, Viswanath et al. (2015) also used the same method to identify two other AHLs (3-OH-C_10_-HSL, 3-OH-C_12_-HSL) synthesized by *V. fischeri*, and accurately classified the AHLs generated by *V. xiamenensis* and *V. proteolyticus* (Viswanath et al., 2015). To date, 18 marine *Vibrio* species were shown to generate AHLs using the TLC-biosensor method (Table 1). Despite the approval of many researchers on its qualitative detection capacity, some drawbacks remain. For example, due to the poor specificity of microbiosensor strains, migrations without matching any of colored or luminous colonization sites of the microbiosensors can easily occur when used in combination with TLC, leading to the inaccurate identification of AHL types (Buch et al., 2003).

Lately, High-Performance Liquid Chromatography tandem Mass Spectrometry (HPLC-MS) with higher sensitivity and specificity was introduced and widely applied for AHLs detection in marine *Vibrio*. HPLC-MS is a physicochemical detection method based on the different retention times of AHLs due to their molecular weights. The AHLs successively enter the mass spectrometer, and their molecular structures are determined based on ion charge-to-mass ratio. HPLC-MS has the LOD in pg level and can provide abundant information on the structures of AHLs. In 1981, Eberhard et al. used HPLC-MS to confirm that 3-oxo-C_6_-HSL was the dominant AHL type produced by *V. fischeri* regulating the bioluminescence of the bacterial community (Eberhard et al., 1981). Kuo et al. (1994) subsequently demonstrated that 3-oxo-C_6_-HSL was superior to C_6_-HSL and C_8_-HSL in inducing the bioluminescence in *V. fischeri* (Kuo et al., 1994). Since the 1990s, the TLC-biosensor method combined with HPLC-MS was commonly used across numerous AHL detection-related studies of marine *Vibrio* for the preliminary screening of AHLs generation and the accurate determination of AHLs types and concentrations (Table 1).

In recent years, Ultra-High-Performance Liquid Chromatography (UHPLC) has been used increasingly as the faster and more sensitive chromatography in detecting AHLs for its great application potentials. UHPLC-MS or UHPLC-Diode Array Detector-Quadrupole Time-Off-Flight Mass Spectrometer (DAD-QTOFMS) even provides accurate identification and quantification of the tested AHLs. Its ultra-high precision, stability and scan quality do not only detect common AHL types but also AHLs with ultra-long acyl side chains (>C_14_) or covalent double bonds (Rasmussen et al., 2014; Table 1). In 2017, Girard et al. first reported the generation of C_14_-HSL in *V. tasmaniensis*, and used UHPLC-MS/MS to confirm the presence of an unsaturated double bond in its acyl side chain (Girard et al., 2017). UHPLC not only overcomes the time-consuming disadvantage of HPLC, but it also greatly increases the types of detectable AHLs, and is therefore a milestone in the study of marine *Vibrio* AHLs.

Moreover, other physicochemical and photochemical methods, such as Gas Chromatograph tandem MS (GC-MS) and Electrospray Ionization tandem MS (ESI-MS), are also widely applied in the detection of marine *Vibrio* AHLs (Taylor et al., 2004; Wang et al., 2013; Table 1). GC-MS analyzes the molecular structures of AHLs based on the differences in adsorption intensity to inert gas and thereby their sequential entrance into the mass spectrometer. However, certain AHL types in the mixture sample may be lost during this process since they are sensitive to temperature change and may degrade during gasification. On the other hand, ESI-MS accurately determines the structure of AHLs via AHL gasification and analysis of the resulting ion fragments. Since AHLs are often a mixture of various types, it is difficult to accurately isolate each of them during gasification and could be missed. Furthermore, other physicochemical methods such as Nuclear Magnetic Resonance (NMR) (Kuo et al., 1994), Infrared Spectroscopy (IS), and Fluorescence Resonance Energy Transfer (FRET)were also used in some studies (Zhang and Ye, 2014). However, these methods are unable to meet the demands of rapid AHLs detection owing to their complicated operation procedures and high requirements on sample preparation. As a result, only few laboratories were able to use these methods for AHLs detection and analysis in marine *Vibrio*, making them unpopular in the research field.

### Immunological approaches

Apart from the aforementioned methods, some studies also attempted detection using immunological approaches. Although several antibodies against AHLs are now available, many limitations still exist. The RS2-IG9 antibody for example, developed against 3-oxo-C_12_-HSL (antigen) from *Pseudomonas aeruginosa* by Kaufmann et al. (2008), has a limited AHL detection range due to its inability to bind other AHLs (Kaufmann et al., 2008). Despite the subsequent emergence of several patented monoclonal Antibodies (mAbs) targeting the homoserine lactone ring or carboxylic acid derivatives on the acyl side chain of AHLs (Janda et al., 2010; Charlton and Porter, 2012; Bhardwaj et al., 2013), many of these mAbs are still under experimental investigation and are therefore not yet applicable for conventional detection.

## Synthesis and regulatory mechanisms of marine *Vibrio* AHLs

### Synthesis of AHLs

**Figure 3:**
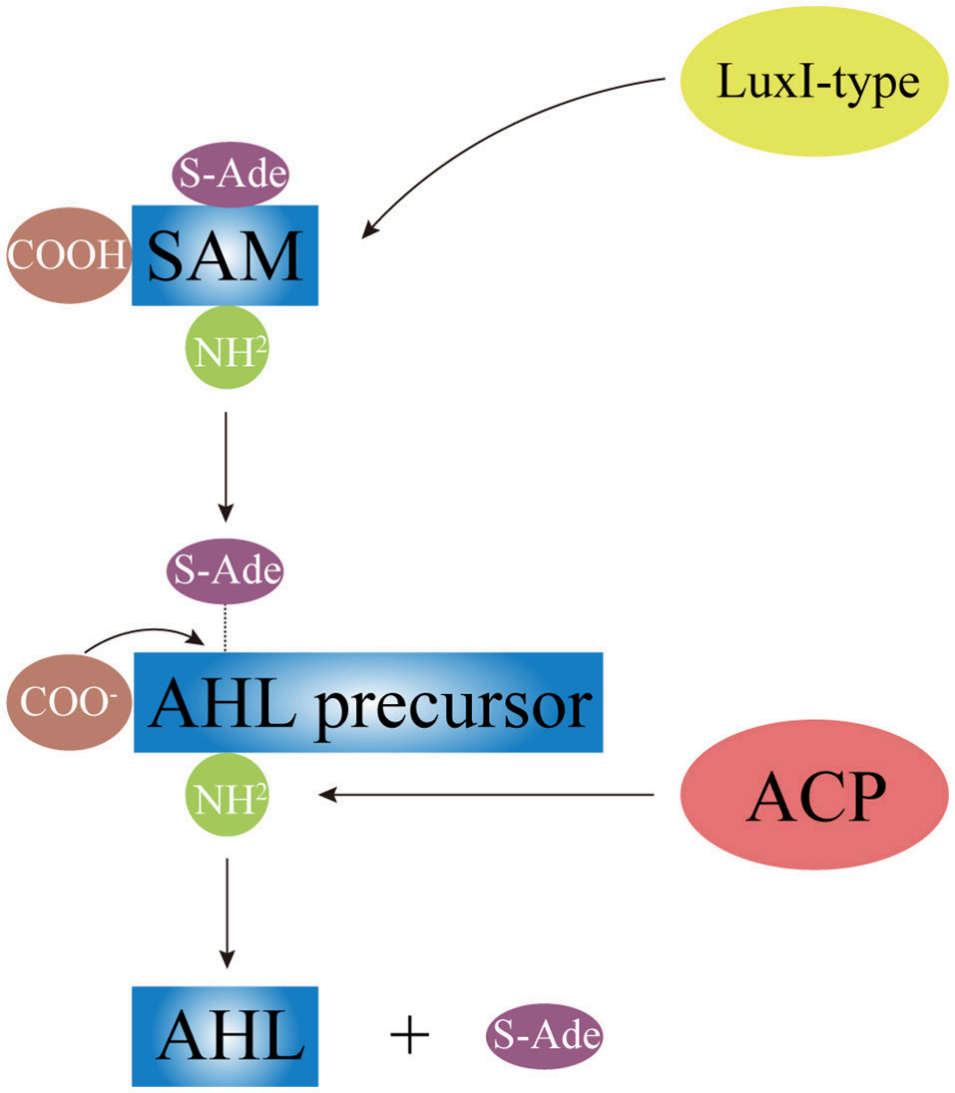
The schematic diagram of AHL synthesis. Under the catalysis of LuxI-type protein, AHL precursor (the HSL ring) is formed via acylating of SAM and removing the S-Ade; the acyl side chain is formed by the transferring of ACP-fatty acyl group derivative onto SAM. SAM, S-Adenosyl-Methionine; S-Ade, methylthioadenosine; ACP, Acyl Carrier Protein.

AHLs can be catalytically synthesized by LuxI homologous proteins (Gilson et al., 1995; Henke and Bassler, 2004; Bruhn et al., 2005; Rasmussen et al., 2014; O'Connor et al., 2015). While some synthtic proteins of AHLs were found in terrestrial bacterial species, such as TraI in *A. tumefaciens* (White and Winans, 2007), RhlI and LasI in *P. aeruginosa* (Brint and Ohman, 1995; Seed et al., 1995), other synthtic proteins of AHLs were present in *Vibrio* species, such as VanI in *V. anguilarum* (Milton et al., 1997), LuxI and AinS in *V. fischeri* (Schaefer et al., 1996; Hanzelka et al., 1999). As Figure 3 shows, LuxI-type proteins first synthesize AHL precursors via the acylation of S-Adenosyl-Methionine (SAM), which removes methylthioadenosine through internal nucleophilic substitution to form the homoserine lactone ring of AHL. Then, Acyl Carrier Protein (ACP)-fatty acyl group derivatives are transferred onto the amino groups of SAM to form acyl side chains with various carbon numbers and chain lengths, which ultimately forms the entire AHL molecule. Difference in the geometric location of the binding site among different LuxI-type proteins determines the status of the third carbon on the acyl side chain, such as saturation (C_3_-H) or oxidation (C_3_-OH, C_3_-oxo), as well as the degree of methylation.

### Regulatory mechanisms of AHLs

As Chapter 1 has mentioned, AHLs are secreted to environment immediately via different secretion pathways after being produced. Short side-chain AHLs are directly released out of the cell upon synthesis while long side-chain AHLs are actively secreted to the environment. Both AHL types are involved in QS signal transduction. There are more than three QS transduction systems existed in *Vibrio*, which present the complexity of diversification and precise regulatory mechanisms in *Vibrio* species (see a review by Milton, 2006). Among all the QS systems, there are two AHL-mediated QS transduction systems in *Vibrio* including the direct “LuxI/R” system and the cascade regulatory system. Of the two the direct “LuxI/R” system first explored in *V. fischeri* is the most known one (Engebrecht and Silverman, 1984). The bioluminescence of *V. fischeri* as an example is the result of LuxR-mediated activation of the LuxCDABE protein, which was also the first QS regulation identified in bacteria. In the LuxI/R QS system, LuxI protein acted as the AHL synthase, and LuxR protein acted as the direct ligand protein of AHLs. The “LuxI/R” system allows bacterial cells to form AHL-receptor complex, which could then bind the functional DNA domain to the subsequent QS related genes (Choi and Greenberg, 1992).

Studies on *V. fluvialis, V. harveyi*, and *V. cholerae* showed that marine *Vibrio* species share similar AHLs regulatory cascades. In an intact AHLs regulatory cascade, the concentration of AHLs increasing to a sensing threshold level of LuxN protein is the key to form AHL-receptor complex in subsequently and to lead a successful QS signal transduction. There are three key molecule types involved in the regulatory cascade of AHLs. The first one is the “two-component” phosphorelay system (Ronson et al., 1987; Parkinson and Kofoid, 1992), where AHLs-sensing LuxN (a cytoplasmic membrane-bound protein) presents as the “input” element and its response regulator LuxO protein as the “output” element (Freeman et al., 2000). LuxN is responsible for sensing AHLs using its “input” domain and for modulating the transmitter activity by changing phosphorylation status of the histidine residue using its transmitter domain (Freeman and Bassler, 1999). LuxO is in response to receive and pass the transmitter signals to the “output” domain by changing phosphorylation status of the aspartate residue. The second one is the Quorum regulatory small RNAs (Qrr sRNAs), and they are in response to degrade the LuxR-type receptor proteins of AHLs via interacting with the chaperone molecule Hfq. The last one is the LuxR-type proteins, which are in charge of activate downstream signaling cascade.

**Figure 4:**
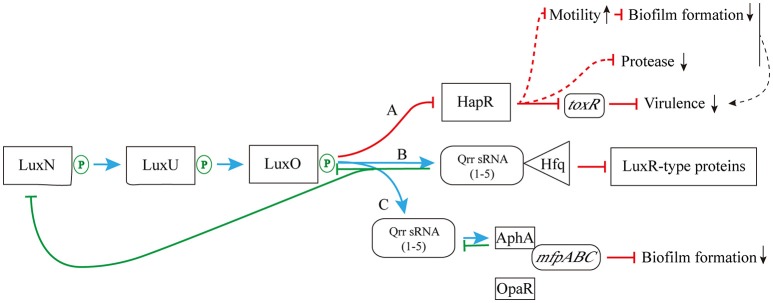
The pathway of phosphorylation for LuxN protein**. (A)** The LuxN phosphorylation inhibits the expression of HapR, and further influences the expression of virulence factor ToxR; **(B)** The LuxN phosphorylation promotes the combination of Qrr sRNA and Hfq, and it constantly degrades LuxR-type proteins; **(C)** The LuxN phosphorylation activates the expression of Qrr sRNAs, allowing competitive combination of transcriptional regulator AphA and membrane fusion operon *mfpABC*, and inhibiting biofilm formation. Blue arrow: positive regulation; red solid T-connector: direct negative regulation; red dashed T-connector: indirect negative regulation; green solid T-connector: direct negative regulation in the feedback pathway; black dashed arrow: indirect positive regulation; green P-circle: phosphorylation; down arrow: weakened expression.

As shown in Figure 4, when the concentration of AHLs is too low to be detected, LuxN presents as kinase, and the autophosphorylation of it occurs normally, activating its downstream transcription factor LuxO via the prior phosphorylation of histidine phosphotransfer protein LuxU (Freeman and Bassler, 1999). This then leads to the expression of Qrr sRNAs (Lilley and Bassler, 2000; Lenz et al., 2004), which sustains the degradation of LuxR-type AHL receptor proteins via interaction with Hfq (Tu and Bassler, 2007). In addition, under the role of two-component phosphorelay system, the phosphorylation of LuxO protein directly promotes the competitive binding of the downstream transcriptional regulators AphA (against OpaR) to the membrane fusion operon *mfpABC* via the activation of Qrr sRNAs expression, which finally inhibits bacterial biofilm formation (Zhou et al., 2013). There are also several feedback mechanisms on Qrr sRNA-related QS cascade (Figure 4; Ball et al., 2017). The increased expression of Qrr sRNAs could directly suppress the expression of LuxO and LuxN protein to maintain the whole dynamic accommodation system (Feng et al., 2015). Coincidentally, the feedback regulation between transcriptional regulator AphA and Qrr sRNAs is almost the same (Rutherford et al., 2011).

**Figure 5:**
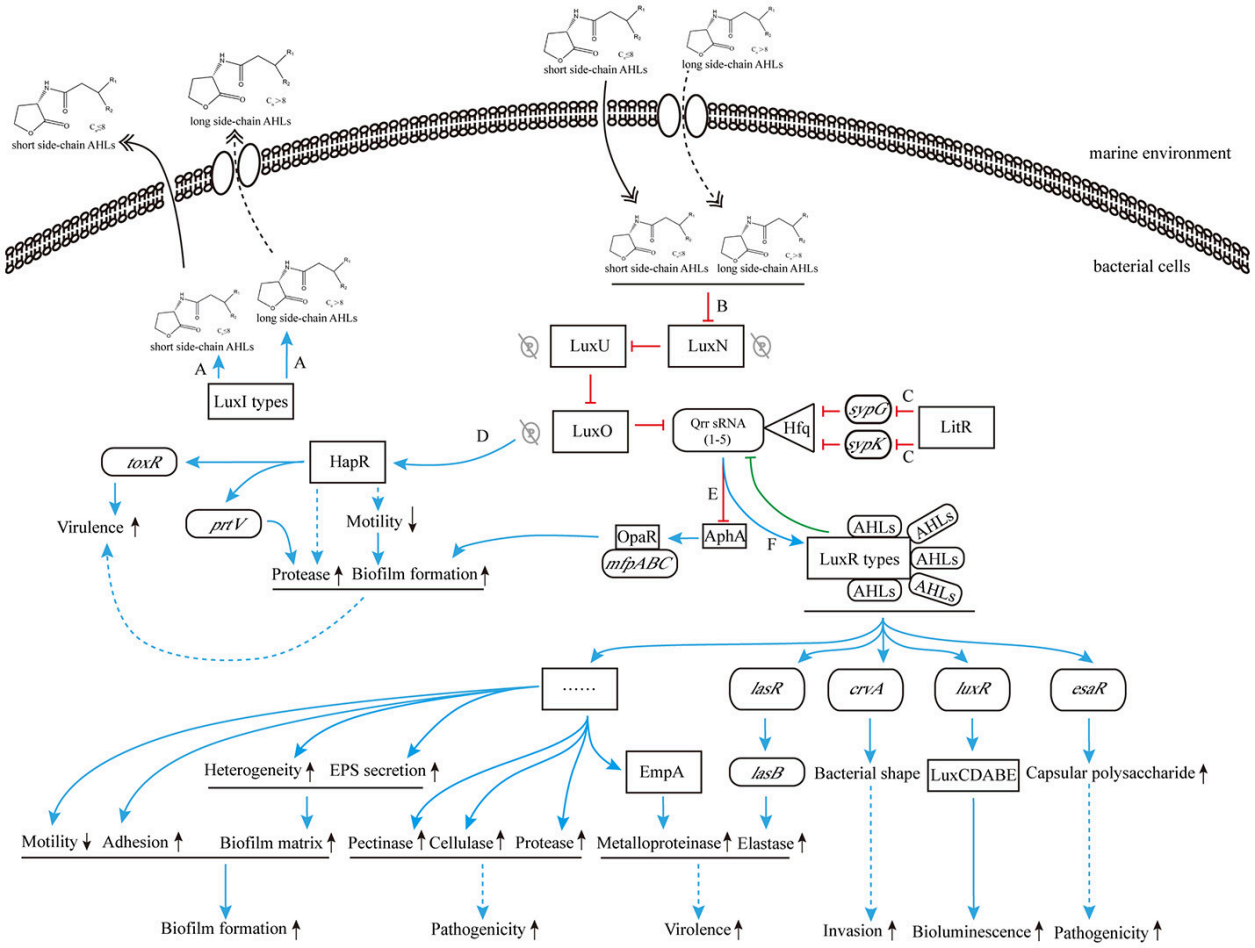
The cascade control mechanism of AHLs produced by marine *Vibrio***. (A)** LuxI-type proteins synthesize and release AHLs to the environment; **(B)** high concentration of AHLs inhibit the phosphorylation for LuxN protein; **(C)** the transcription inhibition of *sypG* and *sypK* by LitR inhibit the combination of Qrr sRNAs and Hfq, and promote the production of LuxR-type protein; **(D)** the inhibition of phosphorylation for LuxN protein removes the surpression of HapR, resulting to direct increased ToxR expression and indirect down regulation of bacterial motility and subsequent increased regulation of biofilm formation and protease production, and promotes bacterial virulence; **(E)** the inhibition of Qrr sRNAs expression is in favor of combining OpaR to *mfpABC*, further increases biofilm formation; **(F)** the AHL-LuxR protein complex activates downstream functional pathways. Blue solid arrow: positive regulation; blue dashed arrow: indirect positive regulation; red T-connector: negative regulation; green T-connector: negative regulation in the feedback pathway; double-headed solid arrow: direct release; double-headed dashed arrow: active transmembrane transport; gray P-circle with a strikethrough: unhappened phosphorylation; up arrow: enhanced expression; down arrow: weakened expression.

On the other hand, when the concentration of environmental AHLs gradually increases to the sensing threshold of LuxN protein, its spontaneous autophosphorylation is inhibited. That in turn inhibits the phosphorylation of downstream proteins (Timmen et al., 2006) such as LuxU protein, which further interferes the phosphorylation of LuxO, leading to the inhibition of Qrr sRNAs expression. As a result, LuxR-type receptor protein is continuously synthesized, and it binds to the acyl side chain of free AHLs to form AHL-receptor transcription complex. The complex regulates the expression of multiple downstream target genes, such as the master regulating gene *luxR* of *V. harveyi* (Van Kessel et al., 2013), the elastase coding gene *lasR* of *P. aeruginosa* (Gambello and Iglewski, 1991), the curvature coding gene *crvA* of *V. cholerae* (Bartlett et al., 2017), and the QS regulon coding gene *esaR* of *Pantoea stewartii* (Ramachandran et al., 2014). Thus, those aforementioned regulations ultimately initiate or silence RNA transcription and protein translation to express related functions (Bassler et al., 1994; Anetzberger et al., 2009; Figure 5).

## Regulatory functions of marine *Vibrio* AHLs

### AHL and biofilm formation

**Figure 6:**
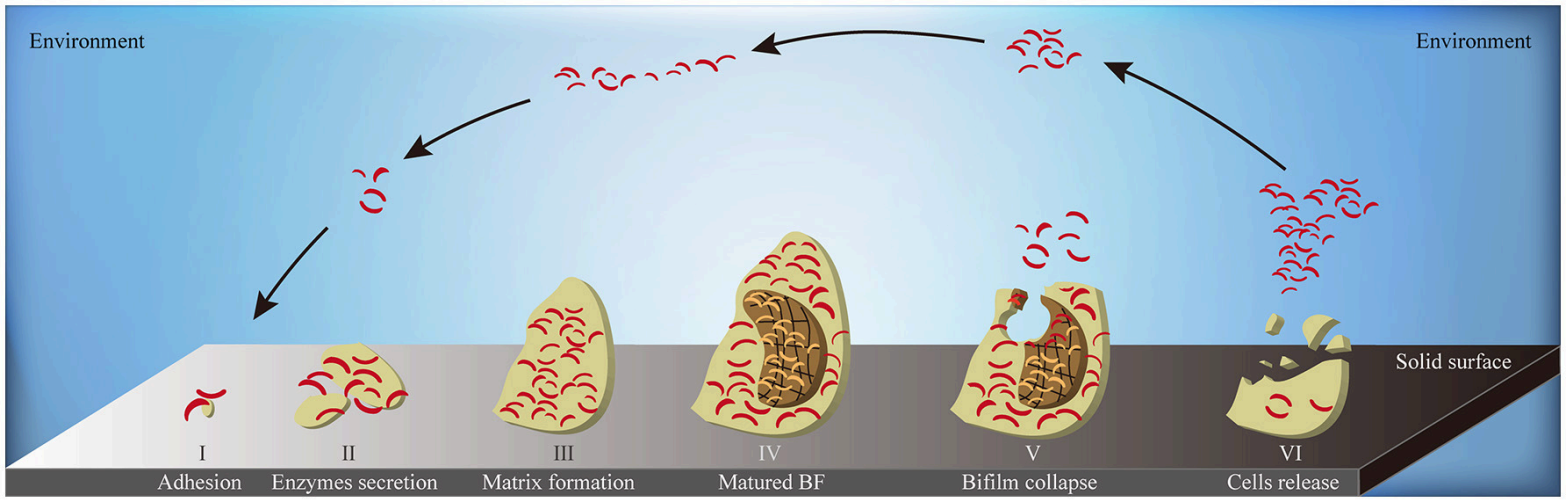
The schematic diagram of biofilm formation. A complete circle of biofilm formation contains: (I) the adhesion of bacterial cells to suitable solid surface; (II) the enhanced secretion of extracellular enzymes; (III) the formation of biofilm matrix; (IV) the formation of matured biofilms; (V) the collapse of biofilm in the middle-end stage; (VI) the release of bacterial cells inside the biofilm to environment in the end stage. BF, biofilm; yellow flat, the entire biofilm; borrow flat, the biofilm matrix; red bend pole, bacterial cells.

When the environmental surface is suitable for bacterial survival, biofilm formation starts from the adhesion of bacterial cells. Along with the enhanced secretion of extracellular enzymes, biofilm matrix builds and biofilm reaches to its mature stage eventually. At the end stage of biofilm formation circle, biofilm starts to collapse, leading to the increased motility of the bacterial cells within the matrix. The collapsing allows bacteria to attach to suitable environmental surfaces, followed by a new period of biofilm formation, including enhanced secretion of extracellular enzymes and formation of biofilm matrix (Figure 6). Biofilm formation is also connected to changes in colony morphology, proliferative metabolism, and drug resistance. Bacteria within the biofilm have significantly slower metabolism and present antibiotic resistance properties. The structure of the biofilm matrix protects bacteria from host cell-mediated or drug-induced phagocytic clearance, allowing the bacteria to evade the host's immune system (Bhardwaj et al., 2013).

Indeed, previous studies have shown that AHLs increases the survival of marine *Vibrio* by regulating key processes of biofilm formation in many ways (McDougald et al., 2006). First, AHLs regulate the excretion of Extracellular Polymeric Substances (EPS) to constitute the cage construction of biofilm matrix. The matrix provides a suitable space for bacterial colonization and stable metabolism. Its porous nature and complex structure allow bacteria cells to hide deeply within the matrix, allowing avoidance of host immune cell-mediated cytotoxicity or phagocytosis and to effectively block the permeation of antibiotics. AHLs such as C_4_-HSL and C_6_-HSL also upregulate the expression of ESP-related genes via binding to AHL receptor proteins, which in turn increase EPS production by forming denser biofilm matrix and reinforced defense barrier (Jamuna and Ravishankar, 2016). Second, AHLs regulate the ability of adhesion or detachment of marine *Vibrio*, allowing the colonization changes for a better adaption to the environment, thus EPS excretion changes in order to quicken or reduce a new period of biofilm formation (Phippen and Oliver, 2015). When the bacteria are in a harsh environment, AHLs enhance bacterial adhesion to adjacent solid surfaces so as to promote clonal proliferation and to speed up the EPS excretion. Once the environmental condition is improved, Qrr sRNAs degrade LuxR-type AHL receptor proteins to reduce *Vibrio* adhesion and enhance their mobility (Phippen and Oliver, 2015), resulting to migration and proliferation of colony to compatible environments. Third, AHLs alter *Vibrio* colony morphology to facilitate biofilm formation. Changes in the colony morphology dynamically regulate the surface area of the biofilm. Compared with a smooth colony, a wrinkled one can effectively increase its surface area to enhance bacterial adhesion to the biofilm. Furthermore, active proliferation of the surface bacteria increases the heterogeneity of the biofilm, which in turns positively regulates its associated functions (Anetzberger et al., 2009). LitR, a transcription inhibitor of the *syp* gene family, inhibits *syp*-mediated transcription of Qrr sRNAs to promote the formation of the AHL-LuxR receptor transcription complex (Miyashiro et al., 2014), which regulates the transformation of smooth to wrinkled *V. salmonicida* colonies, and thus, enhancing biofilm formation (Hansen et al., 2014).

### AHL and bacterial pathogenicity

Bacterial virulence is associated with the strength of bacterial pathogenicity in the host. The invasiveness level and the expression of virulence genes are two critical factors on bacterial virulence, which could exercise either combined effects or solo effects. In most *Vibrio* species, the above-mentioned factors often coordinate with each other. Taken wound infection by *V. vulnificus* as an example, the strong invasive capacity of *V. vulnificus* determines the accurate path and rapid efficiency when entering host bloodstream, and along with *V. vulnificus* proliferation, the subsequent accumulation and regulation of toxin expression via various virulence genes would lead to a high risk of *V. vulnificus*-related death (Lubin et al., 2015). However, as a non-bacteremia *Vibrio* species, *V. cholerae* doesn't cause septicemia but severe diarrhea, acute acidosis and vomiting, which is resulted in the solo-effects of its toxins (Rai and Chattopadhyay, 2014), such as the canonical *Vibrio cholerae* Cytolysin (VCC; He and Olson, 2010).

Substances related to bacterial invasiveness include extracellular enzymes, capsular polysaccharides and other proteins, which play crucial roles in breaking the defense barrier of the host. Bacterial virulence-related proteins encoded by virulence genes could induce the apoptosis of host cells during pathogen infection and lead to the development of various symptoms such as systemic infection and multiple-organ failure. For example, the transcriptional activator ExsA activates the expression of Type III Secretion System 1 (T3SS1 system) and causes disease progression of *V. parahaemolyticus* (Zhou et al., 2008); the pore forming toxin *Vibrio vulnificus* Hemolysin A (VvhA) is an important exotoxin of *V. vulnificus* and causes apoptosis in epithelial cells (Lohith et al., 2015); VCC of *V. cholerae* has potent cell-killing activity and is listed as its prominent membrane-damaging cytolysin (Khilwani and Chattopadhyay, 2015). Early in 1993, the study of Jones et al. (1993) had already revealed the virulence regulation of QS system, and in recent years, more studies have further shown that the expression of virulence-related pathogenic factors of *Vibrio* is strictly regulated by QS system and the environment (Bhardwaj et al., 2013; Lee et al., 2014; Hema et al., 2015; Jung et al., 2015).

Bacterial Extracellular Metalloproteases (BEMPs) are an important type of invasive exocytotic enzymes, and the dependence on the iron acquisition is a key factor for the expression and regulation of BEMPs (Nguyen and Jacq, 2014). BEMPs can be divided into 63 families based on differences in the homologous sequence (Zhang Y. Y. et al., 2017). Elastase is currently the most widely studied enzyme despite no studies or reports of it being produced by *Vibrio*, the production of which is closely related to QS system. As one of the important pathogenic determinants in *P. aeruginosa*, the elastase production is regulated by *lasB* gene, which is activated by the AHL-LasR receptor complex mediated through the binding of the QS signaling molecule 3-oxo-C_12_-HSL to its receptor LasR (Gambello and Iglewski, 1991). During this process, 3-oxo-C_12_-HSL reaching threshold concentration and activating relevant regulatory cascade provide an important intercellular transport pathway for the expression of elastase (Passador et al., 1993).

As reported in the newly published MEROPS database (January 2017)^1^, *Vibrio* species can produce multiple families of BEMPs including M4, M48 and S1. Taken EmpA—a metalloprotease in the BEMP family—as an example, it is a AHL-regulated virulence factor of *Vibrio* (Denkin and Nelson, 2004). Croxatto et al. (2002) have demonstrated that LuxR-type QS transcriptional regulator VanT is required for EmpA expression in *V. anguillarum*. The positive regulation of VanT could enhance EmpA expression, and thereby increase the total secretion of BEMPs from *Vibrio* (Croxatto et al., 2002). QS-mediated regulation of BEMPs has been well exploited in some terrestrial bacteria, such as *lasB*-mediated regulation of elastase via AHL-LasR receptor complex in *P. aeruginosa* (Gambello and Iglewski, 1991; Wei et al., 2015); *Clostridium perfringens* hemolysins CPA and PFO are regulated by the CpAL QS system (Vidal et al., 2015). In *Vibrio* species, HapR protein, a master regulator in *V. cholerae*, has been found to positively regulate *V. cholerae* protease production via upgrading the coding activity of a downstream BEMP related gene *prtV* under the high cell density condition (Figure 5; Nguyen and Jacq, 2014). However, since there are fewer studies on the AHLs-mediated regulation of *Vibrio* BEMPs than there are on common terrestrial bacteria, further supplementary data and investigation are required to elucidate the relevant phenotypes and mechanisms.

Besides the regulation of AHLs on BEMPs, many studies have shown that AHLs participate in the regulation of marine *Vibrio* pathogenicity via regulating other virulence-related proteins. For example, ToxR, a classic *Vibrio* virulence factor encoded by the virulence-related gene *toxR*, is directly regulated by AHLs. ToxR was first discovered in *V. cholerae*, and subsequent studies showed that homologous genes of *toxR* also exist in many pathogenic *Vibrio* species such as *V. parahaemolyticus, V. vulnificus*, and *V. alginolyticus*. When AHLs concentration is below threshold, the regulator LuxO affects the expression of *toxR* gene through inhibition of the production of its upstream protein—HapR, thereby keeping *Vibrio* virulence at a relatively attenuated level (Figure 4). In contrast, when AHLs concentration is higher than the threshold, LuxO is no longer capable of inhibiting the generation of HapR protein, leading to increased toxin synthesis by the *toxR* gene. Meanwhile, bacterial pathogenicity is also synergistically enhanced by the combined regulation of various biological activities, including the down regulation of bacterial motility and subsequent increased regulation of biofilm formation and protease production (Figure 5; Ball et al., 2017).

The curvature determinant protein CrvA, which is another virulence factor, is also regulated by QS signaling molecules. CrvA could alter the vibrioid shape of *V. cholerae* based on the changes occurring in the environment. *V. cholerae* invasiveness increases when CrvA changing the cell from a rod to a bent shape, allowing effective entrance into the host's gut for colonization and proliferation (Bartlett et al., 2017).

Apart from virulence factors ToxR and CrvA, the expressions of various *Vibrio* toxins, such as *Vibrio vulnificus* Hemolysin (VVH) and toxin A (Elgaml et al., 2014), are also directly regulated by AHLs. The synergistic expression of *Vibrio* toxins and virulence factors could enhance bacterial pathogenicity. However, relevant reports on this field remain scarce compared to those done on terrestrial bacteria, which merits further investigation and research.

### Interaction between AHL and host

With an interaction called inter-kingdom signaling (Hughes and Sperandio, 2008), AHLs could modify various types of eukaryotic host cells and modulate host's defense system so as to exert multiple regulatory functions to higher organisms (Hartmann and Schikora, 2012). AHL-mediated regulation of the immune system is a common topic among many current studies.

AHLs directly regulate immune cell proliferation. Taken the signaling molecule 3-oxo-C_12_-HSL as an example, it could inhibit the proliferation of T lymphocytes and human dendritic cells in a dose-dependent manner (10–100 μm; Boontham et al., 2008). Besides, different AHLs have significantly different regulatory efficiency on host cells. AHLs-related comparative study by Gupta et al. (2011) showed that C_4_-HSL and 3-oxo-C_12_-HSL within a certain dose range (1–30 μm) could both inhibit splenic T cell proliferation in mice, while this inhibitory effect could only be detected at high concentration of C_4_-HSL addition alone. In contrast, low concentration of 3-oxo-C_12_-HSL alone was sufficient to inhibit T cell proliferation (Gupta et al., 2011). The study of Gupta et al. (2011) indicates that long side-chain AHLs have better regulatory efficiency than short side-chain AHLs, and the combination of both types has greater inhibitory effect than either AHLs alone.

As well as the regulation of cell proliferation, 3-oxo-C_12_-HSL also affects immune cell survival by inducing apoptosis of neutrophils (Tateda et al., 2003; Li et al., 2010), mast cells and phagocytes in a dose-dependent manner (10–100 μm). 3-oxo-C_12_-HSL could as well activate phagocytosis in human phagocytes via activation of the p38-MAPK pathway, leading to inflammation (Vikström et al., 2005). In the meantime, 3-oxo-C_12_-HSL acts as a chemokine to promote neutrophil migration to the inflammation site and induces host inflammatory response (Zimmermann et al., 2006).

AHLs could alter the host immune response pattern (Rumbaugh et al., 2004). Specifically, high concentration of synthesized AHLs could modulate the immune response of host cells by switching from the Th_1_ immune response, which protects host cells, to the Th_2_ immune response, which is more suitable for bacteria survival (Moser et al., 2002; Hooi et al., 2004). At the same time, these signaling molecules inhibit the activation of Th_1_-type immune response to enhance the AHLs-QS phenomenon (Gupta et al., 2011). During host immune response, other than inducing changes in immune cells, 3-oxo-C_12_-HSL also acts on several cells such as epithelial cells, fibroblasts and lung fibroblasts to synergistically mediate transformation to Th_2_ immune response (Smith et al., 2001, 2002).

In addition to the immune system, AHLs also regulate other cell types such as epithelial cells. 3-oxo-C_12_-HSL is the most commonly studied AHL molecule as it could (1) directly damage the barrier function of intestinal epithelial cells Caco-2 (Vikström et al., 2006), and (2) modify the integrity of epithelial cells via altering tyrosine, serine and threonine phosphorylation in Adherens Junction (AJ) transmembrane protein E-cadherin, cytoplasmic protein β-catenin, Tight Junction (TJ) transmembrane protein occluding, and TJ cytoplasmic protein Zonula Occludens-1 (ZO-1) in a time-dependent manner (Vikström et al., 2009).

## AHLs-related intervention measures

*Vibrio* genus included pathogenic species that are widely found in the marine environment (see a review by Milton, 2006), and their infections cause a series of diseases such as acute gastroenteritis (Shimohata and Takahashi, 2010), septicemia (Horseman and Surani, 2011), and Skin and Soft Tissue Infections (SSTIs; Diaz, 2014). These diseases have acute onset, rapid progression, and may lead to multiple organ failure or even death in severe cases (Janda et al., 2015). As a consequence of increased antibiotic abuse worldwide, the gradual development of Multi-Drug Resistance (MDR) in marine *Vibrio* renders the current measures for *Vibrio* infection less effective, making the search for new anti-*Vibrio* infection measures an urgent focus of research (Elmahdi et al., 2016). As previously discussed in this article, AHLs not only regulate many physiological functions in marine *Vibrio*, but also cause damages to the host cells and immune system, thereby playing a key role in the infection process. With the increased understanding of the regulatory mechanisms of AHLs, blocking key factors in their regulatory pathways and hence inhibiting the downstream effects of AHLs may serve as potential prevention measures and treatments for *Vibrio* infections.

Intervention measures for AHLs-mediated regulation reported in the current literature could be divided into three strategies. The first involves the inhibition of AHLs generation via blocking the synthesis pathway (including the synthesis proteins and the two-component phosphorelay system; Kalia and Purohit, 2011). Specifically, triclosan could inhibit the production of ACP protein by disrupting the chromosomal *fabI* gene, which in turn hinders the function of AHL synthase RhlI and blocks C_4_-HSL synthesis (Hoang and Schweizer, 1999); closantel inhibits the two-component phosphorelay system by altering the structure of histidine kinase sensor “in-put” element (Stephenson et al., 2000), leading to a suppression of AHL synthesis genes (Zhang, 2003). Furthermore, *V. harveyi* R-21446 and *V. harveyi* Fav 2-50-7 isolated from coral-associated microbial colonies could either interfere with the color change of bacterial colony or inhibit biofilm formation of other bacterial groups by blocking AHLs synthesis of the tested bacteria, while the AHL production of themselves was not influenced (Tait et al., 2010; Golberg et al., 2013), and are then considered natural anti-AHL *Vibrio*.

The second intervention type involves the degradation of synthesized AHLs. For example, the AHL-degrading enzyme AiiA produced by *Bacillus* spp. is a lactonase presenting broad spectrum anti-AHL characteristics and displays natural tolerance to acidic environments (Augustine et al., 2010). AiiA inhibits biofilm formation by degrading AHL and blocking its signaling pathway, and significantly reducing the pathogenicity of *Vibrio* in the host, making it a potential AHL inhibitor (Augustine et al., 2010). Further, some higher organisms, such as brine shrimp, have the ability to inactivate AHLs by at least two methods: (1) by providing a highly alkaline intestinal environment, the synthesized AHLs could be hydrolyzed immediately; (2) by producing AHL-inactivating enzymes to reduce the formation of AHL-receptor complex (Defoirdt et al., 2008).

The third-class functions by interfering with AHL-receptor complex formation. Based on the mechanistic pattern of AHL inhibitors, AHL-receptor complex formation could be intervened via four main approaches. (A) Via reducing the binding efficiency of AHL-receptor to its promoter sequence. For example, cinnamylaldehyde and its derivatives block the binding efficiency of transcriptional regulator LuxR-type protein to its promoter sequence, which then affects the expression of the latter, leading to the forming inhibition of AHL-receptor complex, and eventually hinder the downstream regulatory functions of AHLs (Brackman et al., 2008). (B) Via performing the competitive binding to AHL receptor between AHL inhibitor and AHL, and the downstream pathway of gene expression could be eventually blocked. For example, thiazolidinedione analog competitively binds to the binding sites on the amino group or carboxyl group of LuxR protein to block the formation of AHL-LuxR receptor complex (Rajamanikandan et al., 2017), ultimately decreasing the expression of downstream genes. (C). Via changing the structure of AHL receptors. QS inhibitors such as Furanone C-30, which reduces the stability of LuxR receptor and facilitate the structural change of the latter prevent the formation of the AHL-receptor complex (Ren et al., 2001; Lowery et al., 2008). (D) Via modulating AHL receptor-mediated regulation of downstream genes. For example, coumarin significantly reduces LuxR-mediated regulation of downstream genes, and alters the protease activity and hemolytic capacity of *V. splendidus*, resulting in reduced virulence expression (Zhang et al., 2017).

The three major intervention measures target different parts of the AHLs regulatory cascade to inhibit regulation of downstream functions, leading to interference of the infection process and reduced pathogenicity of marine *Vibrio* (Bhardwaj et al., 2013; Chu and McLean, 2016).

## Conclusion and prospects

AHLs are important QS signaling molecules produced by many bacteria genera, especially as the foremost type of QS molecules in a variety of Gram negative bacteria, such as *P. aeruginosa* and *Acinetobacter baumannii* (Smith et al., 2002; Chan et al., 2014). AHLs are not solely restricted to terrestrial bacteria, but are commonly found among marine *Vibrio*. They are involved in many key regulations and play crucial roles in the progress of *Vibrio* infections. With the increased emergence of antibiotic-resistant *Vibrio* species in recent years, studies on QS system have become the new breakthrough for the prevention and treatment of marine *Vibrio* infections.

From the detection methods of AHLs to their production diversity, there are several features about AHLs characterization in *Vibrio* summarized in this article, including types, concentrations, and dominance alteration. Among these features of *Vibrio*, an article by Buchholtz et al. (2006) discussed about the dominant AHL change in *V. anguillarum* along with differing environments, which so far has only been reported once. Yet, is this dominance changing of AHLs only happening in *Vibrio*? Or, is there any close relationship between this phenomenon and *Vibrio* adaption to different environments? There is still no further research continuing with these hypotheses. Currently, from AHLs regulating functions to AHL-related QS prevention strategies, most studies focus on common terrestrial pathogens such as *P. aeruginosa* and other bacteria especially on the interaction between AHLs and host cells, while the fewer studies in *Vibrio* are still on their exploration stage based on the similar researches in terrestrial bacteria. This means either a stagnate or a slow-moving forward in this field, which is expected to be seen for a breakthrough in the future.

Since the research direction is somewhat limited as above-mentioned, further studies will be required to determine other specific AHLs-related functions and regulatory mechanisms that may be present in *Vibrio* species. Therefore, expansion of research on the generation, regulation and relevant functions of AHLs in marine *Vibrio* has great application potentials and deserve further in-depth investigations.

## Author contributions

JL and LZ conceptualized this review. KF and LZ designed the frame structure of this review based on the idea. JL and KF organized the original writing of this review. CW and KQ confirmed the logic validity of this review. FL and LZ proofread the paper. JL and KF contributed equally to this work. All authors discussed the conclusion and commented on the manuscript.

### Conflict of interest statement

The authors declare that the research was conducted in the absence of any commercial or financial relationships that could be construed as a potential conflict of interest. The reviewer YT and handling Editor declared their shared affiliation.

## Footnotes

^1^https://www.ebi.ac.uk/merops/index.shtml

## Abbreviations

N-AHL: N-Acyl Homoserine Lactone
QS: Quorum Sensing
AI: AutoInducer
AI-1: AutoInducer-1
AI-2: AutoInducer-2
AIP: AutoInducing Peptide
MS: Mass Spectrometry
X-Gal: 5-bromo-4-chloro-3-indole-β-D-galactopyranoside
LOD: Limits of Detection
TLC: Thin Layer Chromatography
LSAC: Liquid-Solid Absorption Chromatography
HPLC: High-Performance Liquid Chromatography
UHPLC: Ultra-High-Performance Liquid Chromatography
DAD: Diode Array Detector
QTOFMS: Quadrupole Time-Off-Flight Mass Specrometer
GC: Gas Chromatography
ESI: Electrospray Ionization
NMR: Nuclear Magnetic Resonance
IS: Infrared Spectroscopy
FRET: Fluorescence Resonance Energy Transfer
mAb: monoclonal Antibody
SAM: S-Adenosyl-Methionine
ACP: Acyl Carrier Protein
Qrr sRNA: Quorum regulatory small RNA
EPS: Extracellular Polysaccharides
T3SS1 system: Type III Secretion System 1
BEMPs: Bacterial Extracellular Metalloproteases
VvhA: *Vibrio vulnificus* Hemolysin A
VCC: *Vibrio Cholerae* Cytolysin
AJ: Adherens Junction
TJ: Tight Junction
SSTIs: Skin and Soft Tissue Infections
MDR: Multi-Drug Resistance.
